# High Frequency of Cell-in-Cell Formation in Heterogeneous Human Breast Cancer Tissue in a Patient With Poor Prognosis: A Case Report and Literature Review

**DOI:** 10.3389/fonc.2019.01444

**Published:** 2019-12-19

**Authors:** Banzhan Ruan, Zubiao Niu, Xiaoyi Jiang, Zhuo Li, Yanhong Tai, Hongyan Huang, Qiang Sun

**Affiliations:** ^1^Department of Oncology, Beijing Shijitan Hospital of Capital Medical University, Beijing, China; ^2^Department of Biology, Hainan Medical University, Haikou, China; ^3^Laboratory of Cell Engineering, Institute of Biotechnology, Beijing, China; ^4^The Fifth Medical Center of the Chinese PLA General Hospital, Beijing, China

**Keywords:** cell-in-cell formation, cell cannibalism, entosis, heterogeneity, breast cancer, poor prognosis

## Abstract

Cell cannibalism is a unique pathological phenomenon that has been observed at low frequency in a variety of human tumor samples (<0.5%), including breast cancer. Cannibalistic cells typically form cell-in-cell (CIC) structures characterized by enclosure of one cell or more by another, mediating a novel type of cell death “entosis,” which was proposed as the type IV cell death. A large number of CIC structures are generally associated with malignant transformation and progression, and they are believed to be primed by and form among heterogeneous cells. However, there is currently no *in vivo* evidence from human tumor samples. In this case report, covering a 37-year-old female breast cancer patient, we observed considerable heterogeneity and proliferative activity (>70% Ki-67 positivity) in her breast cancer cells, accompanied by high frequency of CIC formation (~6%) and poor prognosis. We consider this a typical example of cell cannibalism, supporting a role of heterogeneity in cell-in-cell formation and malignant progression. It may serve as a pretest basis for further investigations of cell-in-cell biology and breast cancer treatment.

## Introduction

Breast cancer tissues display profound heterogeneity, which is important for clinical diagnosis and therapy. Cell cannibalism is a unique pathological phenomenon that has been observed in various types of human tumors, including breast cancer (1). Cannibalistic cells typically form cell-in-cell (CIC) structures, characterized by enclosure of cells by another one, and this affects patient prognosis (2, 3). CIC structures have not only been found in human tumors but also in animal tumors (4), suggesting that CIC is a general malignant phenomenon across species. Recent advances have shown that CIC structures play important roles in not only tumor evolution and genome instability but also embryonic development and immune homeostasis (5).

Multiple mechanisms, such as entosis, emperitosis, and homotypical cell cannibalism, have been proposed to promote CIC formation either homotypically between tumor cells or heterotypically between lymphocytes and tumor cells, which generally leads to the death of the internalized cells (5, 6). The death of the engulfed cells was believed to be executed non-autonomously by the engulfing cells, so CIC structures are believed to mediate a novel type of cell death process that parallels the existing cell-autonomous death processes apoptosis, necrosis, and autosis (7). Entosis is the best studied mechanism underlying the formation of CIC structures between tumor cells, and it is driven by polarized actomyosin that is compartmentalized by p190A RhoGAP recruited to E-cadherin-mediated adherens junction (8, 9). Factors regulating either actomyosin or adherens junctions have turned out to more or less affect entotic CIC formation (10, 11), and these effects can be induced by either matrix detachment, aberrant mitosis, or glucose starvation. Although these three inducers initiate entosis via distinct molecular mechanisms, the signal transduction converges eventually onto RhoA-ROCKs-regulated actomyosin (5, 11), suggesting that the cytoskeleton plays a pivotal role in controlling entosis. Activation of entosis probably serves as a competition mechanism that targets abnormal less fit cells for internalization and subsequent death to promote the selection of fitter cell clones. In this way, entosis was implicated in the evolution of heterogeneous tumors (6, 12, 13).

Since matrix detachment is a strong inducer of entotic CIC formation (14), tumor cells in effusion fluids are likely to form a high frequency of CIC structures (2). As for solid tumor tissues, these types of cannibalistic structures were generally identified in low frequency (<0.5%) (15), probably due to complex cell adhesions that prevent asymmetric cell internalization. Here, we reported an unusual case of breast cancer patient whose tumor was highly heterogeneous and contained a considerable amount of complex CIC structures (~6%), which may be related to active cell proliferation and may be involved in unfavorable prognosis.

## Case Report

**Figure 1:**
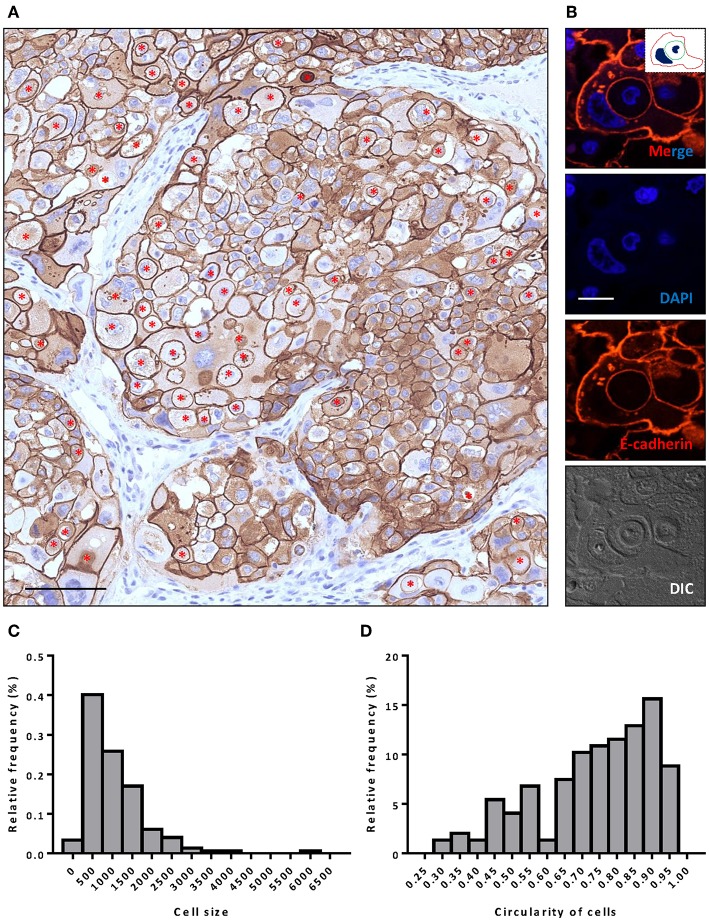
Extensive CIC formation in heterogeneous cancer tissue. **(A)** Representative image for HER-2 staining. Inner cells of CIC structures are indicated with red asterisks. Scale bar: 100 μm. **(B)** Representative image for a typical CIC depicted with E-cadherin staining. Inserted picture of the top merged image is a schematic cartoon for the indicated CIC structure. Scale bar: 20 μm. **(C,D)** Histogram plots of cell size **(C)** and cell circularity **(D)** for **(A)**.

**Figure 2:**
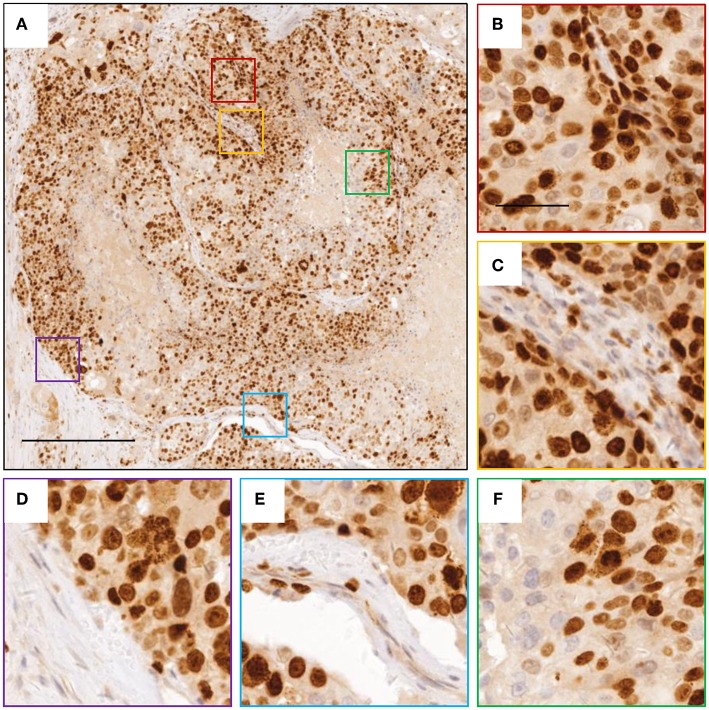
Images of Ki-67 staining indicating active cell division. **(A)** representative image for breast cancer tissue with Ki-67 staining. **(B–F)** zoomed in images for boxed regions in **(A)**. The scale bars are 400 and 100 μm, respectively.

A 37-year-old female was diagnosed with breast cancer 2 years ago. She had no family history. A tumor was found in the left chest, with lung metastases, but none were found in the axillary lymph nodes, so it was initially diagnosed as invasive ductal carcinoma of grade 3. Computerized tomography (CT) of the chest identified single tumor (4.9 × 3.1 × 4.5 cm). While the focal dermal layer of inner skin was involved, the nipple and striated muscle within mammary glands did not. Biopsy tissues displayed a large degree of cellular heterogeneity with tumor cells varying significantly in shape and size (Figure 1) and a high rate of Ki-67 positivity indicating active proliferation (Figure 2). Immunohistochemistry indicated the expression of HER-2 (3+), E-cadherin (+), Ki-67 (>70%), CK5/6 (+), EGFR (Weak +), and Top-IIα (+, 60%).

**Figure 3:**
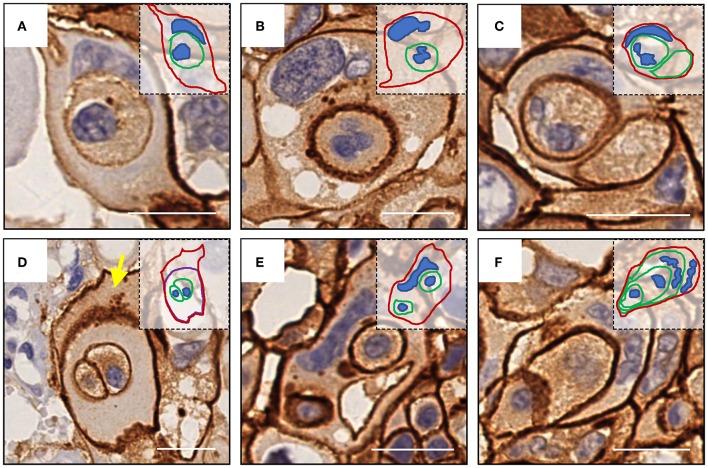
Complex morphologies of CIC structures. **(A)** One cell was internalized. **(B)** abnormal nucleus of the outer cell. **(C)** Two cells were internalized, and the nucleus of one of them was missing. **(D)** The cell enclosing two cells was inside of another cell (yellow arrow) without nucleus. **(E)** Two cells were internalized, and the nucleus of the outer cell was deformed. **(F)** Sequential internalization of three cells. Inserted pictures are schematic cartoons for the indicated CIC structures, respectively. Scale bar: 20 μm.

Like E-cadherin, HER-2 staining labeling cell boundary depicts a number of unique structures morphologically resembling CIC structures (Figures 1A,B). The overall frequency reached 6% of all tumor cells counted, which is pretty rare for solid tumors. The structures identified were complex. While most of them contained one cell (Figures 3A,B), some contained two or more (Figures 3C–E), which may have been caused by multiple internalization events or a single internalization event followed by mitotic cell division. Furthermore, some cells seemed to internalize sequentially to form superposition structures (Figure 3F). The outer cell nuclei were generally abnormal (Figures 3B,D) or irregular with some being split or multiple (Figures 3C,E,F), indicating aneuploidy or multi-ploidy, consistent with the report that CIC could induce aneuploidy by blocking cytokinesis of the engulfing cells (16).

The patient had an unfavorable prognosis. After diagnosis, she was given four cycles of TXH (T = docetaxel, X = capecitabine, H = herceptin) rescue chemotherapy, and then two cycles of XH treatment due to hand–foot syndrome. However, the disease progressed. Two cycles of vinorelbine plus trastuzumab were then applied, and tumor volume kept increasing. Next, palliative resection was performed, followed by 26 weeks of paclitaxel and lapatinib. *Staphylococcus aureus* infection occurred in the peripherally inserted central catheters. After anti-infection treatment, the patient reached a stable condition which was maintained for 5 weeks after 3 weeks of paclitaxel. Due to poor tolerance, therapy was changed to etoposide plus lapatinib. Nearly 4 months later, chest CT showed lung metastasis, and some lesions got larger in the following 2 months. Finally, gamma knife treatment (DT5600Gy/8f) was performed.

## Discussion

The roles of CIC in human cancers had been controversial (6), while the initial studies proposed a tumor suppressive role based on its nature of cell death, subsequent researches also identified tumor promotive functions for CIC-mediated engulfment. This discrepancy was resolved recently by the concept of cell competition (12, 17). Heterogeneous tumors generally contain multiple clones that compete with each other for limited space and nutrients. During the early stage, CIC death limited tumor growth. By CIC-mediated engulfment, the winner tumor cell clones that harbor oncogenic mutations such as *Kras*V12 (12) repetitively internalized and outcompeted those that were less malignant, leading to a slowing of tumor growth. CIC-induced aneuploidy endows the winner cells more opportunity to acquire new mutations and malignant phenotypes, such as metastasis. As a result, the malignant winner clones with oncogenic mutations eventually populate the tumor tissues and undergo distant metastasis during the late stage of cancer (18). Accordingly, high frequency of CIC structures precedes malignant transformation and progression, which is consistent with the case reported here, in which the tumor kept growing and progressing to lung metastasis despite sustained therapy.

Whereas, heterogeneities within tumor clones drive CIC formation, the process has been shown to be complex and genetically controlled (19). E-cadherin-mediated adherens junctions bring cells together, and set up asymmetric RhoA activity to drive cell internalization (8, 9) with the assistance of optimal membrane cholesterol and lipids (20) and the inflammatory cytokine IL-8 (21). Durgan et al. (22), and our unpublished work as well, identified cell division as a potent inducer of entotic CIC formation, the mechanism might also work in this case as the tumor cells are undergoing active division as indicated by >70% Ki-67 positivity. A review of the limited literature on CIC formation in breast cancer (Table 1) showed that CIC structures were also frequently associated with active cell proliferation (3, 22, 24) and, to an extent, cellular heterogeneity (15, 16, 24, 25); and the frequencies of CIC structure, although difficult to compare due to the different types of calculation, span a wide range from presence (24, 25, 27) to 6% in this study.

**Table 1:** Reports on CIC in human breast carcinoma.

**Authors**	**Year**	**Case**	**Cancer type**	**Sample**	**CIC**	**Main finding**	**References**
Fujii et al.	1986	1	Invasive ductal carcinoma	Nipple discharge	Present	Malignant epithelial cells and cell clusters were observed	(23)
Abodief et al.	2006	50	Ductal breast carcinoma	Tissue sections	<0.7%^*^	Cell cannibalism index associates with high grade of breast carcinoma	(15)
Overholtzer et al.	2007	4	Primary human breast carcinomas	Pleural effusions, tissue sections	2.5%^#^	CIC invasion mediates nonapoptotic death of internalized cells	(14)
Krajcovic et al.	2011	15	High grade or metastatic breast carcinoma	Pleural effusions, tissue sections	Present	CIC formation blocks outer cell cytokinesis to promote aneuploidy	(16)
Almeida and Rotta	2015	1	Metastatic breast carcinoma	Cerebrospinal fluid	Present	Cell cannibalism in the cerebrospinal fluid of a patient with metastatic breast adenocarcinoma	(24)
Durgan et al.	2017	75	Invasive ductal carcinomas	Tissue microarray	17/mm^2^^#^	Mitosis can drive cell cannibalism through entosis	(22)
Kinoshita et al.	2018	25	Squamous cell, apocrine, invasive ductal carcinoma	Tissue sections	Present	Cannibalism are useful indicators for the differential diagnosis of SCC of the breast	(25)
Zhang et al.	2019	148	Ductal breast carcinoma	Tissue microarray	14/mm^2^^*^	Subtyped CIC structures are independent prognostic markers that impact patient survival	(3)
Ruan et al.	2019	1	Invasive ductal carcinoma of grade 3	Tissue section	6%^*^	High frequency of CIC formation in heterogeneous breast carcinoma	(26)

# Highest CIC structure.

* *Mean CIC structure*.

In conclusion, the case reported here is a typical example of cell cannibalism, and its pathological features fit well current studies on CIC formation and functional implications, supporting the role of heterogeneity in CIC formation and malignant progression. It may serve as a pretest basis for further investigations on CIC biology and breast cancer treatment.

## Methods

### Tissue Processing and Staining

Tissues were fixed in 10% (v/v) neutral phosphate-buffered formalin and then embedded in paraffin. Tissue sections of 5 μm were routinely de-paraffinized following standard Xylene-Ethonal method after being baked in 65°C for 1.5 h. Antigen retrieval was performed in citrate acid buffer by the microwaving method for 15 min after boiling. Then, the slides were blocked with 5% (w/v) BSA for 1 h at room temperature followed by incubation with the primary antibodies overnight at 4°C. For immunofluorescence (IF) staining, Alexa Fluor 568 anti-mouse secondary antibody (1:500) was applied for 1 h at room temperature before mounted with Prolong Gold antifade reagent with DAPI (Invitrogen). For immunohistochemistry (IHC) staining, HRP-conjugated secondary antibodies (1:2000) were applied for 1 h at room temperature before developed by DAB reagent.

### Image Capture and Processing

IHC slides were scanned with NanoZoomer S60 (Hamamatzu Photonics) and analyzed with NDP.view 2.6.13 (Hamamatzu Photonics). Confocal images were captured on Ultraview Vox spinning disc confocal system (Perkin Elmer) on a Nikon Ti-E microscope and processed with Volocity 6.0 software. Cell size and morphology were analyzed using NIS Elements 4.5 software (Nikon). Briefly, images in JPG format were opened by the Element 4.5 software, and the irregular shape tool in the Object Catalog was selected and applied to individual cells, following the marking of cell contours. The information on cell size and circularity could be exported for further analysis and plotting with GraphPad Prism 6.01 (GraphPad). The carton print of images were made by curve painting in PowerPoint (Microsoft).

## Data Availability Statement

All datasets generated for this study are included in the article/supplementary material.

## Ethics Statement

The studies involving human participants were reviewed and approved by Institutional Review Board of Affiliated Hospital of Academy of Military Medical Sciences [Beijing]. The patients/participants provided their written informed consent to participate in this study.

## Author Contributions

QS, HH, and BR prepared the figures and wrote the manuscript. ZN performed histological examination with the help of XJ. ZL and YT collected clinical data. BR performed immunohistochemistry analyses. All authors read and approved the final manuscript.

### Conflict of Interest

The authors declare that the research was conducted in the absence of any commercial or financial relationships that could be construed as a potential conflict of interest.
